# Semantic Terrain Segmentation in the Navigation Vision of Planetary Rovers—A Systematic Literature Review

**DOI:** 10.3390/s22218393

**Published:** 2022-11-01

**Authors:** Boyu Kuang, Chengzhen Gu, Zeeshan A. Rana, Yifan Zhao, Shuang Sun, Somtochukwu Godfrey Nnabuife

**Affiliations:** 1 Centre for Computational Engineering Sciences (CES), Cranfield University, Cranfield MK43 0AL, UK; 2 Supply Chain Research Centre, Cranfield School of Management, Cranfield University, Cranfield MK43 0AL, UK; 3 Centre for Aeronautics, Cranfield University, Cranfield MK43 0AL, UK; 4 Centre for Life-Cycle Engineering and Management, Cranfield University, Cranfield MK43 0AL, UK; 5 College of Aviation Engineering, Civil Aviation University of China, 2898 Jinbei Road, Dongli District, Tianjin 300300, China; 6 Geo-Energy Engineering Centre, Cranfield University, Cranfield MK43 0AL, UK

**Keywords:** rover autonomy, visual localization, open dataset, image processing, terrain annotation

## Abstract

*Background*: The planetary rover is an essential platform for planetary exploration. Visual semantic segmentation is significant in the localization, perception, and path planning of the rover autonomy. Recent advances in computer vision and artificial intelligence brought about new opportunities. A systematic literature review (SLR) can help analyze existing solutions, discover available data, and identify potential gaps. *Methods*: A rigorous SLR has been conducted, and papers are selected from three databases (IEEE Xplore, Web of Science, and Scopus) from the start of records to May 2022. The 320 candidate studies were found by searching with keywords and bool operators, and they address the semantic terrain segmentation in the navigation vision of planetary rovers. Finally, after four rounds of screening, 30 papers were included with robust inclusion and exclusion criteria as well as quality assessment. *Results*: 30 studies were included for the review, and sub-research areas include navigation (16 studies), geological analysis (7 studies), exploration efficiency (10 studies), and others (3 studies) (overlaps exist). Five distributions are extendedly depicted (time, study type, geographical location, publisher, and experimental setting), which analyzes the included study from the view of community interests, development status, and reimplementation ability. One key research question and six sub-research questions are discussed to evaluate the current achievements and future gaps. *Conclusions*: Many promising achievements in accuracy, available data, and real-time performance have been promoted by computer vision and artificial intelligence. However, a solution that satisfies pixel-level segmentation, real-time inference time, and onboard hardware does not exist, and an open, pixel-level annotated, and the real-world data-based dataset is not found. As planetary exploration projects progress worldwide, more promising studies will be proposed, and deep learning will bring more opportunities and contributions to future studies. *Contributions*: This SLR identifies future gaps and challenges by proposing a methodical, replicable, and transparent survey, which is the first review (also the first SLR) for semantic terrain segmentation in the navigation vision of planetary rovers.

## 1. Introduction

Recent planetary exploration accomplished encouraging achievements and keeps attracting various community interests because of the promotions of advances in robotics, artificial intelligence, computer vision, sensor, and space science. For example, the Zhurong and Perseverance rovers landed on Mars in 2021 and carried out many scientific missions [1,2], while Canada, Japan, Saudi Arabia, and Russia announced their ambitious lunar (Moon) rover projects [3,4,5]. The planetary rover is an essential platform for planetary exploration, widely involved in various scientific activities, including geological exploration [6], planetary history investigation [7], extraterrestrial water resource exploration [8], unknown environment perception [9], life exploration [10], and Mars sample return [11]. However, terrains are significantly complicated and hazardous in these activities, possibly bringing rovers into the “mission-ending scenario” [12]. For example, Spirit and Opportunity Mars rovers are stuck with rugged terrains [13,14], the loose and granular terrains can cause their wheels to slip and sink [15], and the large rocks can block their paths. Therefore, terrain recognition is important for planetary exploration because terrains have considerable differences in inaccessibility. 

The planetary rover is a sophisticated research platform. The vision-based technology in this review only limits the image or video signal captured by the pinhole camera, which is the review scope of this study. The sensors used for the navigation of rover autonomy can be classified into active and passive [16], and vision-based technology mainly refers to passive sensors. Active sensors (such as radar, laser scanner [17], structured light [18], time-of-flight (ToF) [19], etc.) are sensitive to environmental changes, heavy-weighted, have high energy consumption, and large-sized [16,20,21]. Millimeter-wave radar relies on the millimeter wave, which has a wavelength between 1 and 10 mm. The ejected wave can be reflected back when the obstacle is approached, and the radar can use the reflected signal to estimate the distance. However, the resolution of radar is relatively low. Thus, this study only surveys passive sensors. Lidar (laser scanner) measures the distance between the lidar sensor and the target to estimate the 3D structure of the target using the energy magnitude, frequency, and phase of the reflected spectrum. Lidar can be used in autonomous driving for distance estimation, 3D reconstruction for achieving accurate point clouds, and information fusion to improve 3D estimations. Structured light displays a pattern on the target surface and estimates the surface structure using the distortion of the pattern. ToF has a similar principle to radar, while only the millimeter wave becomes the light. The pinhole camera model is a typical example of passive sensors [22]. Compared with active sensors, the pinhole camera is lightweight, has low energy consumption, and small-size. 

The vision-based navigation represented by the pinhole camera is important, which can bring a superior solution with lightweight, low energy consumption, and small-size for rover autonomy. The pinhole camera model can be divided into mono- and multi-camera systems [23,24]. The mono-camera system acquires single images, while the multiple-camera system is associated with image pairs. Furthermore, the visual signal (image, image-pair, or video) is essential for planetary rover navigation. It is attractive and challenging to extract, understand, and deliver the information from visual signals efficiently. Semantic segmentation is essential in visual understanding [25], and vision-based rover autonomy relies on semantic understanding [13]. Image is a digit matrix for machines, while visual understanding segments the pixels into pixel clusters associated with categories. Therefore, this study specifies the scope of the candidate studies to the camera system-based works, corresponding to the data format of the image, image-pair, or video.

Terrain semantic segmentation is a highly interdisciplinary topic where recent developments have greatly influenced deep learning and computer vision. For example, references [26,27,28] adopt the image processing-based method (superpixel and threshold), references [29,30,31] apply the unsupervised clustering machine learning algorithms (K-mean cluster or SVM), and references [11,13,32] adopt some advanced neural networks (Deeplabv3, Mask-R-CNN, or U-Net). However, a comprehensive discussion is lacking, challenging future studies on the choice of quantitative metric, available dataset, or qualitative demonstration. This study reviews these studies with a methodical, replicable, and transparent survey for future research and the corresponding community.

This study conducts a rigorous systematic literature review of semantic terrain segmentation in the navigation vision of planetary rovers. The authors of [33,34] recommend the systematic literature review as the approach for comprehensively implementing “Evidence-based Software Engineering” (EBSE). The EBSE emphasizes an evidence-based review strategy to ensure methodological rigor [34]. The systematic literature review is a methodical, replicable, and transparent survey that achieves robust and broad conclusions and implications by summarizing, synthesizing, and evaluating individual studies [35]. The necessity of conducting this systematic literature review includes the following four aspects: Firstly, the topic of this study is a specific application, while the narrative review tends to survey a broad topic [36,37]. Secondly, the unified and precise criteria are the basis for ensuring that study selection is comprehensive and fair, while the narrative review does not require such criteria [36,37]. Thirdly, this study made a rigorous statistical analysis of the *Included Study*, while a narrative review focuses on qualitative discussion [36,37]. Finally, it is essential to draw conclusions based on the statistical analysis to guide future research, rather than relying on the authors’ subjective analysis and judgment in the narrative review [36,37].

The contributions of this study can be summarized as below:

1. This study provides a methodical, replicable, and transparent survey for semantic terrain segmentation in the navigation vision of planetary rovers. It provided robust and broad conclusions and implications for communities by summarizing, synthesizing, and evaluating individual studies.
2. This study discussed and summarized existing research results through a systematic literature review and accordingly proposed potential gaps and challenges for future study.
3. As far as the authors are aware, this study is the first review and the first systematic literature review on the topic of semantic terrain segmentation in the navigation vision of planetary rovers.

The structure of this study is as follows: Section 2 describes the method of this systematic literature review. Section 3 depicts the results of the review process. Section 4 discusses the research questions proposed in Section 2 and the limitations. The conclusion is then presented in Section 5.

## 2. Method

This study adopts a rigorous systematic literature review following the guideline in references [33,34] and uses references [34,38,39,40,41] as examples. This study has two objectives: Firstly, to identify, classify, and summarize current studies. Secondly, to analyze and locate the potential gaps and opportunities for future studies.

**Figure 1:**
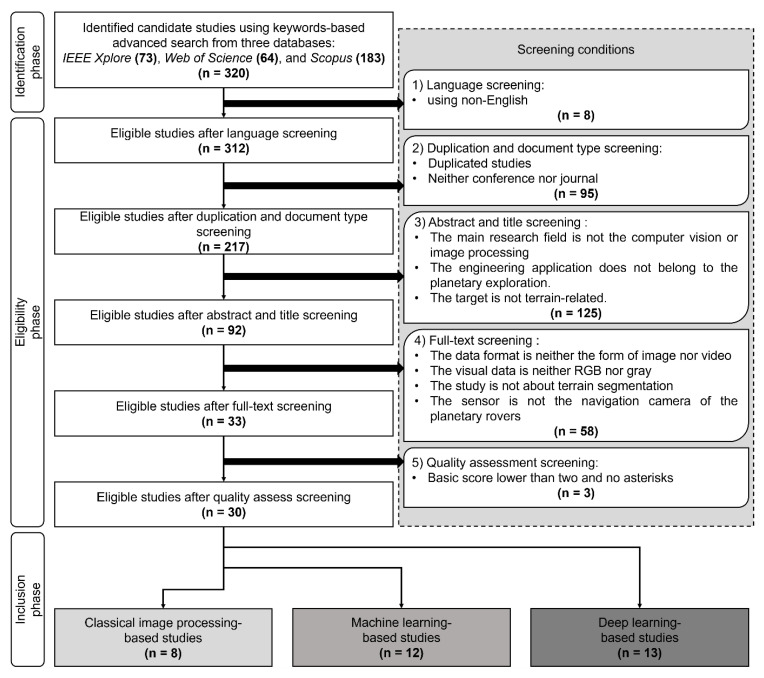
The flow diagram of this systematic literature review. The search process has been separated into three phases, “*Identification phase*”, “*Eligibility phase*”, and “*Inclusion phase*”. The screening follows the inclusion and exclusion criteria depicts in Section 2.3. The “n” in each frame refers to the number of studies for the corresponding action or category.

Figure 1 depicts the flow diagram for conducting this systematic literature review. The search process is divided into three phases, the identification phase, the eligibility phase, and the inclusion phase. Firstly, the identification phase identifies the candidate studies from the three databases. Secondly, the eligibility phase applies the screening conditions according to the inclusion and exclusion criteria. The quality assessment is conducted as the last step of the eligibility phase. Finally, the inclusion phase classifies the *Included Study* into three categories. The italic “*Included Study*” specifically represents the studies selected after four screenings and one quality assessment.

This study defines three categories to classify the Included Study, and they are represented using the italic “Classical Image Processing-based study”, “Machine Learning-based Study”, and “Deep Learning-based Study”. The classical image processing-based study does not apply to the learning process. It is noteworthy that this study considers the learning process to be the iteration to achieve a valid semantic segmentation. For example, neural networks use iteration to update the computation graph [42], and SVMs apply iteration to minimize loss [43]. Therefore, the classical image processing-based study refers to the study whose semantic segmentation model does not require such iteration. The machine learning-based study and deep learning-based study both require iteration in the learning process. Although deep learning is generally considered a sub-discipline in machine learning [44], this study separates the deep learning-based study into an individual category using the iteration kernel. Deep learning has recently begun to dominate semantic visual segmentation [45,46], and Section 4.1.4 also indicates that the community’s attention to the deep learning-based study increased significantly. Specifically, the deep learning-based study refers to the study that uses the programmable iteration kernel, while the machine learning-based study refers to the study that applies the unprogrammable iteration kernel. For example, the neural network can be significantly different using different layer combinations and structures [42], while the SVM can only choose a few options (linear, polynomial, and sigmoid) [43].

### 2.1. Research Question

Table 1 describes the research questions (RQ) addressed by this study. The key research question (KeyRQ) is divided into six sub-research questions (SubRQs), sub-research question 1 (SubRQ1) to sub-research question 6 (SubRQ6). This study aims to determine the current benefits and the future potential that computer vision and deep learning have and can bring to semantic terrain segmentation. The KeyRQ is framed with the guideline of the PICOC criteria (population, intervention, comparison, outcome, and context) [33]. In the KeyRQ, The “navigation vision of the planetary rovers” refers to the population, the “computer vision and artificial intelligence” refers to the intervention, the “What...?” represents the comparison, the “achievements” stands for the outcome, and the “semantic segmentation” refers to the context.

1) Sub-research question 1 (SubRQ1) addresses the importance of terrain segmentation in planetary explorations from motivation and impact. This study addresses the importance of terrain segmentation by summarizing the undergoing projects and clarifying current research interests and motivations worldwide.
2) Sub-research question 2 (SubRQ2) then explores the targeting terrains in current research and the corresponding reasons. It is important to locate the research targets and corresponding reasons in current research because planetary exploration is a highly unstructured environment.
3) Sub-research question 3 (SubRQ3) describes the data from the perspective of the sensor and data format, which addresses the research scope from the aspect of hardware and collected data.
4) Sub-research question 4 (SubRQ4) discusses the existing solutions and characteristics. This study identifies the drawbacks of current research and achieves inspiration for potential improvement.
5) Sub-research question 5 (SubRQ5) depicts the data availability from the perspective of data science.
6) Sub-research question 6 (SubRQ6) addresses the evaluation metrics and corresponding state-of-the-art performance.

### 2.2. Search Strategy

The identification phase includes the candidate studies into the scope of the systematic literature review as comprehensively as possible. Three databases have been used, IEEE Xplore [47], Web of Science [48], and Scopus [49], and the identification phase was completed in May 2022. There are two reasons for using these three databases: First, these three databases cover most of the relevant literature. Second, these three databases all provide advanced search functions based on logical operators, making retrieval comprehensive and efficient. The specific search method is the keyword search in the advanced search function.

The search commands are designed the same in the three databases. The search command consists of four parallel searching conditions using the “AND” logic operator, and these four conditions correlate to the PICOC composition of the KQ (see Section 2.1). It is noteworthy that “*” in the searching command refers to a wildcard. For example, search command “terrain*” can refer to “terrain”, “terrains”, and “terrain”, plus other spellings.

(i) The intervention, comparison, and outcome represent the specific details in studies, which can only be achieved via full-text screening. Thus, this study left them to be investigated in the full-text screening.
(ii) The context is divided and correlated into two conditions.
  (ii-a) The first condition scopes the candidate study into terrain-targeted, corresponding to the “terrain” in the context. It is noteworthy that rocks and sky are also included in the terrain category because terrain is a vast concept. The authors of [13,50] discuss the important semantic terrain in the navigation vision of planetary rovers, claiming that various types of rocks play a critical role in planetary exploration missions. Rock can be any concept related to rocks, such as bedrock, rocks, etc. The sky also refers to the non-sky area (ground) and the “skyline”. Skyline refers to the boundary between sky and non-sky regions.
    Therefore, the first condition is searched for in the scope of the title, abstract, and keywords, and the search command is “terrain* OR *rock* OR sky*”.
  (ii-b) The second condition scopes the candidate study into segmentation-related, corresponding to the “segmentation” in the context. Some studies only work on terrain classification or path planning, which is not considered the proper candidate study in the search strategy.
    The second condition is searched for in the scope of the title, abstract, and keywords, and the searching command is “segment*”.
(iii) The “Population” in KeyRQ is also divided and correlated into two conditions.
  (ii-a) The third condition scopes the candidate study into planetary exploration-related, corresponding to the “planetary rovers” in the population. Some studies address autonomous car driving or moon detection, which is not considered a valid candidate study in the search strategy.
    The third condition is searched for in the scope of the title, abstract, and keywords, and the search command is “planetary OR mars OR lunar OR Martian OR moon”.
  (ii-b) The fourth condition scopes the candidate study into image or video data format, corresponding to the “navigation vision” in the population.
    The fourth condition is searched in the scope of the full-text, and the search command is “image* OR vision OR visual”.

### 2.3. Inclusion and Exclusion Criteria

The exclusion and inclusion criteria refer to the “Eligibility phase” screening conditions in Figure 1. The eligibility phase uses five screenings to select the *Included Study* from the candidate studies. There are 320 candidate studies in the identification phase, consisting of 73 from IEEE Xplore, 64 from Web of Science, and 183 from Scopus.

1) Language screening: exclude non-English documents.

Non-English documents are prone to errors in reading and comprehension. Eight candidate studies were screened, and 312 eligible studies were left.

2) Duplication and document type screening: remove duplicated documents and keep only conference or journal publications.

There were 95 candidate studies screened, and 217 eligible studies were left.

3) The abstract and title screening: only screened according to the abstract and title
  a) The screened study is not in computer vision or image processing scope. Computer vision is the “Intervention” in the PICOC criteria [33] for the KeyRQ.
  b) The studied scenario is not planetary exploration. Planetary exploration is related to the “Population” in the PICOC criteria [33] for the KeyRQ.
  c) The target is not related to terrain.
    This abstract and title screening removed 125 studies, and 92 studies were left.
4) The full-text screening: screened according to the full-texts, and the following four types of results are removed:
  a) The data format is neither image nor video. Image and video are two typical data formats from the passive visual sensor, while other data formats are very different from what this study addressed.
  b) The image or video neither consisted of color nor grayscale format. Some studies use disparity images or infrared images, which are not in line with the objectives of this review.
  c) The study is not semantic terrain segmentation. Terrain segmentation refers to the “Context” in the PICOC criteria [33] for the KeyRQ.
  d) The camera is not the navigation vision of the planetary rovers. Some studies use satellite or telescope images.
    This step removed 58 results, and 33 results were left.
5) The quality assessment screening: screened according to the quality assessment result.

Section 2.4 designed the quality assessment criteria.

### 2.4. Quality Assessment

This study conducted the quality assessment following the guidance in reference [51], and the quality assessment criteria can be found in Appendix A of reference [51]. Five elements are assessed, the “theory robustness”, the “implication for practice”, the “methodology, data supporting arguments”, “generalizability”, and the “contribution plus a short statement summarizing the article’s contribution” [51]. There are five levels to indicate the assessment results:

- “0” level stands for “Absence”, which refers to “the article does not provide enough information to assess this criterion”.
- “1” level stands for “Low”.
- “2” level stands for “Medium”.
- “3” level stands for “High”.
- “Not applicable” level stands for “This element does not apply to the document or study”.

### 2.5. Data Collection

The following information was extracted from every study involved in the search process. The records of the extracted information were described in brackets. The “or”, “and”, and “others” refer to single choice, multiple-choice, and omitted details, respectively.

- The title
- Digital object identifier (DOI)
- The authors
- The country of the corresponding author
- The publication time by year
- Publication type (conference, journal, or book)
- The source databases (IEEE Xplore, Web of Science, and Scopus)
- Main research topics (computer vision, image processing, planetary exploration, semantic segmentation)
- Studied targets (terrain, rock, soil, craters-related terrain, hazard/safe area, obstacle, horizon/skyline, shadow, sample tube, sky/ground, slippage, wheel sinkage, unknow, and others)
- Data format (color image, gray image, infra-red spectrum image, or depth image)
- The research data source (customized dataset, specific public dataset, or unknown)
- The number of images in the research data source
- Sensor type (whether it is the rover navigation camera)
- Camera model (stereo camera, mono camera, or unknown)
- Classification of the research method according to Figure 1 (classical image processing-based study, machine learning-based study, or deep learning-based study)
- The details of the corresponding solution (edge detection, Canny operator, Deepv3+ model, superpixel, support vector machine, and others)
- The applied metrics and corresponding quantitative results
- The qualitative results (yes or no)
- The summary of research questions (see Section 2.1 and Section 4.1)

The data collection takes the suggestions from references [33,34] and used references [34,38,39,40,41,52,53] as examples.

## 3. Results

### 3.1. Search Results

Thirty studies are identified from the database search to be included for analysis. Table 2 summarizes the details of the *Included Study*, while the quantitative results are listed in Table 3.

Regarding the first row, the “Ref.”, “Year”, “F_data_”, “S_cate_”, “S_data_”, “N_img_”, “Quali.”, “Quanti.” refer to the reference index, publication time by year, data format, classification of the research method according to Figure 1, research data source, number of images in the research data source, qualitative results, as well as applied metrics and corresponding quantitative results, respectively. Regarding the tabular content, the “IP.”, “ML.”, “DL.”, and “N/A” refer to the classical image processing-based study, machine learning-based study, deep learning-based study, and not-applicable.

The search results are depicted using five distributions. Section 3.1.1 uses the distribution by time (year) to indicate the trend of interest in history. Section 3.1.2 addresses the distribution by type to evaluate the research progress of semantic terrain segmentation because the conference and journal studies refer to different research statuses. Section 3.1.3 analyzes the distribution by geographical countries, considering that the planetary exploration is usually supported by government projects. The distribution by the publisher is addressed in Section 3.1.4 to identify the interest in different communities. Finally, Section 3.1.5 discusses the distribution by the experimental setting, which is essential for reimplementation for future studies.

#### 3.1.1. Distribution by Time

**Figure 2:**
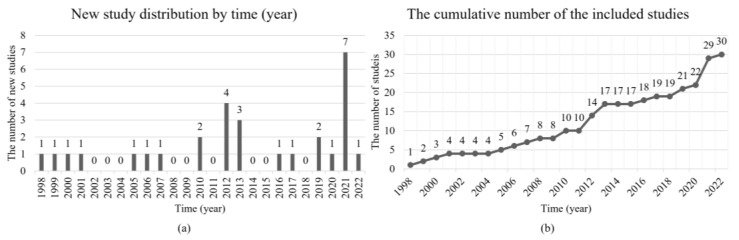
The statistics of the new studies in the *Included Study*. (**a**,**b**) Refer to the increasing and cumulative number of the *Included Study*.

The distribution by time (year) indicates the increasing trend of the new *Included Study* per year, which shows that interests change in the community (see Figure 2). Figure 2a shows the new studies per year, and the publication time can be found in Table 2. Figure 2b presents the cumulative number of the *Included Study*. Firstly, there was an intensive increase between 2010 and 2013, and nine studies were published. Chinese Moon landing (Chang’E [77]) and lunar rover (Yutu [78]) projects contribute to the increase of studies during this period. Five corresponding affiliations of the nine studies are from China (refs. [14,26,28,29,71]) and two from Japan (refs. [60,61]). Secondly, the newly *Included Study* attaches the maximum number compared throughout the period in 2021. The reason may be related to the two successful Mars rovers from the United States of America (USA) and China. The time distribution shows the incremental attention to the semantic terrain segmentation, and planetary exploration projects closely influence the new studies.

#### 3.1.2. Distribution by Study Type

**Figure 3:**
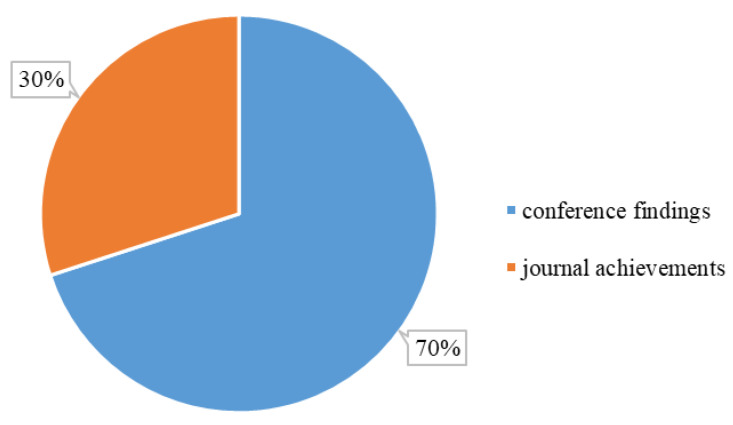
The distribution by study type in the *Included Study*. The “conference findings” and “journal achievements” refer to the studies published in the conferences and journals, respectively.

This section analyzes the distribution of the *Included Study* by type. The conference usually presents the primary findings, and the journal addresses the systematic results. The conference findings can be considered the previous step of journal achievements [79]. However, references [79,80] claim that conference findings in computer science tend towards a rapid communication approach instead of only primary findings. The peer-review process for conferences is usually faster than journals, which can flexibly respond to rapid developments. As an interdisciplinary topic, many computer science techniques are widely utilized in semantic terrain segmentation (for example, image processing, machine learning, and deep learning). Figure 3 indicates the distribution by study type (conference findings or journal achievements). The number of conference findings is double that of journal achievements, indicating that semantic terrain segmentation in the navigation vision of planetary rovers is constantly developing.

#### 3.1.3. Distribution by Geographical Location

Planetary exploration is an expensive activity usually sponsored by government investment [81]. Table 4 depicts the distribution by geographical location, and location is determined according to the affiliations of the corresponding author. The USA contributes the most (more than 40%) to the *Included Study*, and China is second (about 30%). The *Included Study* is only originated from three continents in the Northern Hemisphere (North America, Europe, and Asia) with regards to the distribution by continent. Interestingly, seventy percent of Asian studies were published between 2010 and 2013, which might be influenced by the Moon landing activity [77,78]. Furthermore, although not many studies are directly affiliated with Europe, they bring significant impacts considering their extensive and international cooperation [82].

#### 3.1.4. Distribution by Publisher

**Figure 4:**
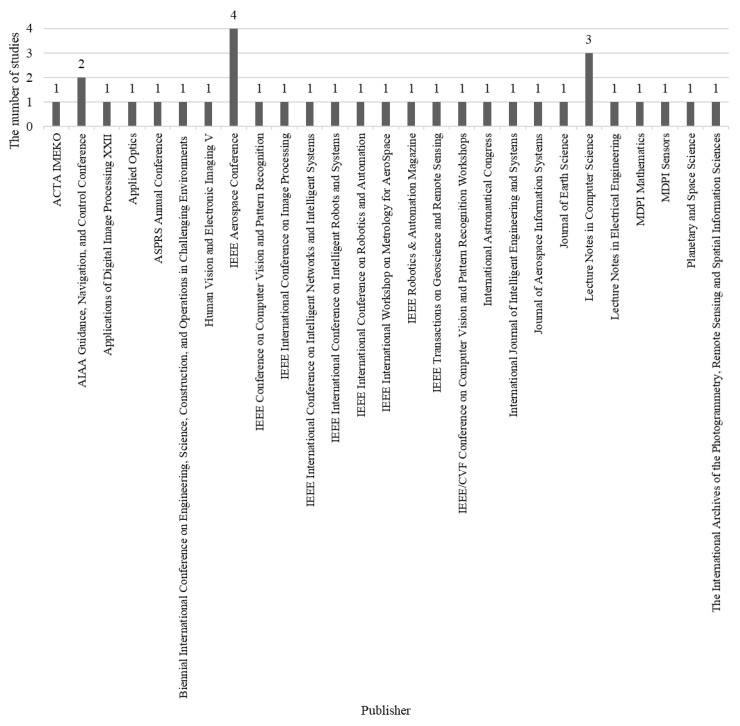
The distribution by publisher in the *Included Study*.

Figure 4 illustrates the distribution by publisher. The publisher has its reader and author groups—whom the studies are published by indicate the interests of the correlated community. The IEEE aerospace conference archives contain the highest number (four studies), while the *Included Study* is divided between approximately 27 publishers. Figure 4 indicates that the interested community is widely divided into various subjects, including, but not limited to, robotics, artificial intelligence, computer vision, navigation, remote sensing, environment, automation, mathematics sensor, and aerospace.

#### 3.1.5. Distribution by the Experimental Setting

**Figure 5:**
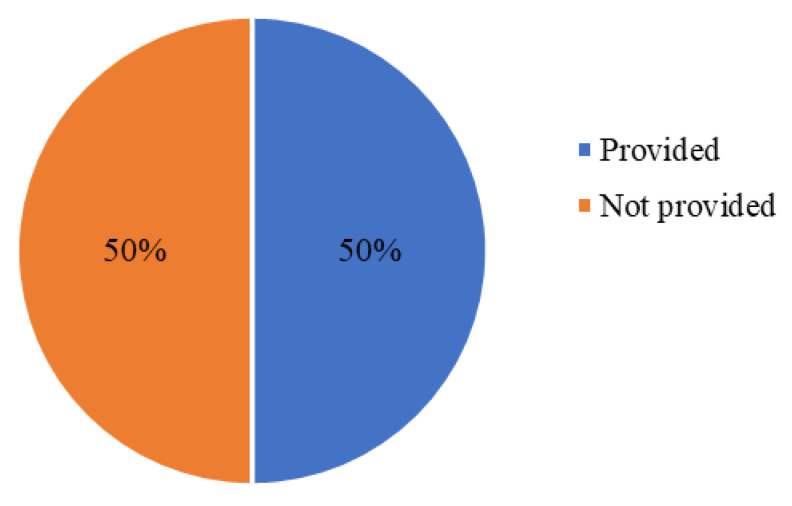
The distribution by the experimental setting.

Reimplementation is a common challenge for current research [83]. Reimplementation consists of duplicating the proposed solution in the corresponding study, which is essential for justifying the contribution and novelty in future studies. A common approach for justifying the contribution and novelty is to compare the proposed results with existing solutions in a comparable experimental setting. Furthermore, some studies may contribute to transferring the existing solutions from one scenario to another. Providing the significantly helpful experimental setting for reimplementation, which can also improve the reliability by notifying readers of the preconditions of the results. This study takes the experimental setting as either the hardware or the software conditions. Figure 5 indicates the ratio between experimental settings provided and not provided in the *Included Study*., and Table 5 depicts the reference list of the distribution in Figure 5. Notably, fifty percent of the *Included Study* do not provide the experimental setting, which can cause significant difficulties in reimplementing the corresponding solution and decrease the reliability of results.

### 3.2. Quality Evaluation

The quality evaluation applies the quality assessment criteria in reference [51] (depicted in Section 2.4), and the quality assessment results are depicted in Table 6. The quality evaluation only considers studies with the “Sum in points” of less than eight as the included study. Therefore, the quality evaluation excludes the studies of references [77,84,85].

## 4. Discussion

This section describes the potential research gaps and challenges through the KeyRQ and SubRQs. Firstly, Section 4.1 extensively discusses the six SubRQs. Then, Section 4.2 depicts the answer to the KeyRQ. Finally, the limitations of this study are addressed in Section 4.3.

### 4.1. The Answer to the Sub-Research Questions

#### 4.1.1. SubRQ1: Why Is Terrain Segmentation Important for Planetary Explorations?

Semantic terrain segmentation is a basic function for planetary exploration missions that supports the building of many practical applications in practice. Table 7 divides the field of application in the *Included Study* into four categories: navigation, geological analysis, exploration efficiency, and other particular purposes (finding water or returning Mars samples).

More than half of the *Included Study* addressed navigation. Current planetary rovers rely mostly on remote control from Earth bases, while an autonomous navigation system only works under minimal conditions and periods [13]. As the mission distance increase, the planetary rovers increasingly require safer, more real-time, and more accurate navigation systems [29,86,87]. However, according to the experience of Earth-based navigation, intelligent navigation relies highly on semantic information [13]. Therefore, exploring semantic segmentation technology for planetary rovers is essential. The navigation category covers the broad scopes of regular rover navigation, path planning, obstacle avoidance, and autonomous navigation.

Geological analysis is another critical mission for planetary exploration, and Table 7 indicates that about 20% of the *Included Study* focused on geological analysis. For example, studying geographies from other planets can help us understand the history and development of the Earth [28]. Some studies analyze geological information to trace the existing water on the planet [8]. However, most geological analyses are conducted manually through remote communication [28]. The data acquisition speed has increased dramatically and is faster than the manual analysis speed [28]. Some geological information is sequenced, which might occur in different spots and timestamps within the rover missions [28]. Thus, it is easy to miss important geological information during planetary rover operations [59]. One solution is to analyze the data automatically, and another is to select the important data and filter the unimportant data for human researchers. Both of them rely strongly on semantic information. The “important” should correspond to the specific mission, for example, rocks for reference [30] and water for reference [8].

#### 4.1.2. SubRQ2: What Targets Does Current Research Pay Attention To?

**Figure 6:**
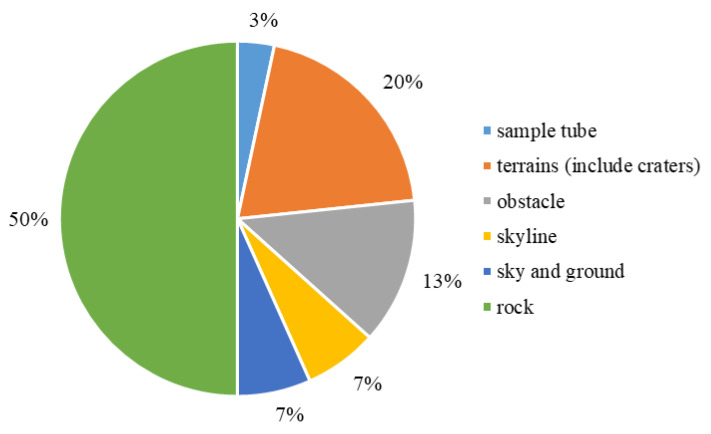
The ratio distribution by the target in the *Included Study*.

The studied targets in the *Included Study* involve the sample tube, terrains, obstacle, skyline, sky (and ground), and rock (the sample tube is the target of reference [11] in the *Included Study*). Figure 6 illustrates the ratio of these studied targets, and Table 8 depicts the reference list to corresponding targets in Figure 6. Terrains refer to studies focusing on multiple instead of one terrain. Obstacles target obstacle avoidance, which only concerns whether the path could pass the target. Skyline is a similar target to the sky (and ground). Sky and ground are two common semantic labels in planetary exploration, and the boundary between sky and ground refers to the skyline. The skyline can be used to identify the rover’s location by matching the skylines and measuring the rover’s position. Sky and ground regions can also be used for further processes. Rock is a very common target in the *Included Study*, and 54% of studied targets are rocks.

Although 54% of the *Included Study* targeted rock, rock segmentation is still challenging. Rocks have significantly different appearances, and it is challenging to use unified properties to identify the background and rocks [59]. However, identifying the rocks in the navigation vision is essential for path planning and geological analysis. The shape, weathering, and location of rocks contain information on the environmental properties and historical processes. Therefore, it is necessary to segment rocks to identify their geological properties [59].

#### 4.1.3. SubRQ3: What Have Visual Sensors Been Applied to for Obtaining Data?

Sensors can be classified into two categories, exteroceptive and proprioceptive sensors [13,88]. The exteroceptive sensors conduct localization using the data from the surrounding environment. The exteroceptive sensors are not suited for planetary rover autonomy, which includes global navigation satellites, range sensors, vision sensors, 3D to 2D perspective projection, and vehicular network sensors. (1) Planetary rover exploration is a global navigation satellite system-denied scenario [89]. (2) The range sensors (like laser scanners and radar) are heavy in weight with high energy consumption, which can increase the load of the planetary rover. (3) The 3D to 2D perspective projection relies on the knowledge of the camera parameters and transformation matrix, which is only measurable when the image is in focus. Planetary exploration is a complex environment with challenging illumination, noise, and reflection conditions, which decreases the reliability of 3D to 2D perspective projection. However, the vision sensor (like a camera system) is lightweight, has low energy consumption, and has robust working requirements, and this study only addresses vision-based sensors (camera system).

The proprioceptive sensors rely on internal measurements (such as velocity and steering angle). The proprioceptive sensors contain vehicle motion sensors and inertial sensors. The proprioceptive sensors are used as a data fusion to support the localization task. However, this review concentrates on the aspect of visual semantic segmentation, while the proprioceptive sensors are not within the scope.

**Figure 7:**
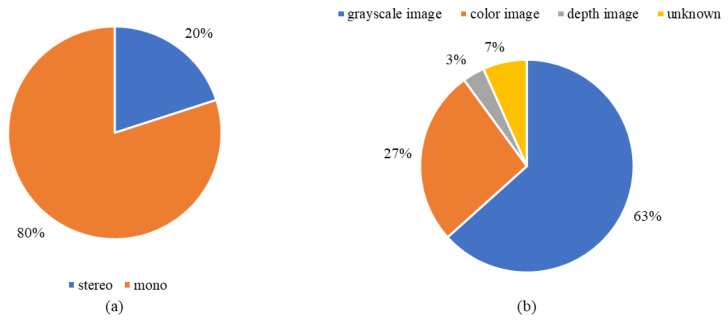
The distribution by camera model and image format in the *Included Study*. (**a**) Refers to the ratio of the monocular camera model in the *Included Study*. (**b**) Refers to the distribution by image format in the *Included Study*.

Therefore, the sensors are limited to the stereo camera and monocular camera, which are passive optical camera systems. Figure 7a indicates the distribution of sensors in the *Included Study*, and Table 9 depicts the study of the distribution by camera model as in Figure 7a. The stereo image pairs refer to the data obtained from the stereo camera system, while the monocular images refer to the data from the monocular camera. Furthermore, 80% of monocular images are utilized in the *Included Study*, and only 20% apply stereo image pairs. The multi-camera system has higher power consumption and device weight, and this distribution indicates that the navigation system of the planetary rovers would most likely prefer a mono-camera system instead of a stereo.

Figure 7b and the “F_data_” column in Table 2 further analyze the distribution by the image type in the *Included Study*, and Table 10 indicates the study of the distribution by image format in Figure 7b. The grayscale image refers to only one channel image, while the color image refers to the three-channel image (red, green, and blue channels). Depth image comes from the stereo camera, which can be calculated from the disparity image. Sixty-three percent of the *Included Study* used the grayscale image because most information of the visual signal can be well-contained using grayscales. Depth image usually requires considerable memory and computation power, and only 3% of the studies applied it.

#### 4.1.4. SubRQ4: What Solution Does Current Research Have?

This section further classifies the methods in the included study as the classical image processing-based study, machine learning-based study, and deep learning-based study. The classical image processing-based study refers to the traditional methods. This review classifies any study without applying machine learning or deep learning method as the classical image processing-based study. The attribute for machine learning and deep learning is learning, which corresponds to the iteration process. In other words, the image processing method has no iteration for the purpose of segmentation. Deep learning is usually considered a subject of machine learning. However, since Hinton proposed the deep belief networks (DBN) in 2006 [90], the deep network-based method has accomplished significant achievements in various sectors [91,92,93,94]. Therefore, this review separates the deep learning-based study as an individual category. This review distinguishes the machine learning-based study or deep learning-based study through the operation kernel of the solution. If the kernel is requires programming, then the study belongs to the deep learning-based study. Otherwise, it belongs to the machine learning-based study.

The “S_cate_” and “Algorithm” columns in Table 2 list the statistical results of the method classification and the specific algorithm used in the corresponding *Included Study*. The “ip”, “ml”, and “dl” in the “Solution category” column refer to the *Classical Image Processing-based Study*, *Machine Learning-based Study*, and *Deep Learning-based Study*, respectively. It is noteworthy that the “SIFT”, “SVM”, “TDEL”, and “UNet” in the “Algorithm” column refer to the spatial invariant feature transform, support vector machine, template dilatation edge linking [71], and U-shaped network [74], respectively.

**Figure 8:**
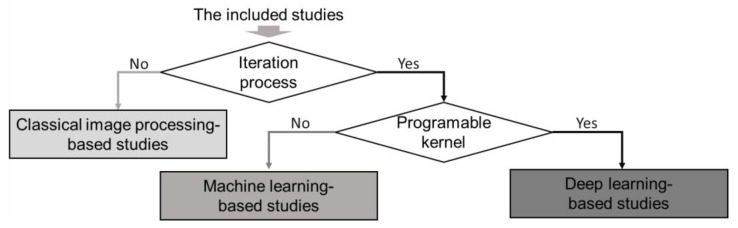
The classification rules of the proposed three categories for the included study (the classical image processing-based study, the machine learning-based study, and the deep learning-based study).

Figure 8 illustrates the relationships and classification rules among the three proposed categories for the *Included Study* in Section 2. Thus, Figure 8 firstly applies the discriminant condition of “whether the *Included Study* applied the iteration process for the terrain segmentation purpose?” The “No” studies go to the “*Classical Image Processing-based Study*”, and the “Yes” studies introduce the second discriminant condition. The second discriminant condition is “whether the iteration kernel of the *Included Study* is programable?” The “No” studies go to the “*Machine Learning-based Study*”, and the “Yes” studies go to the “*Deep Learning-based Study*”. It is noteworthy that the classification rules for the proposed three categories in Figure 1 and Figure 8 are only valid under the conditions of this review.

**Figure 9:**
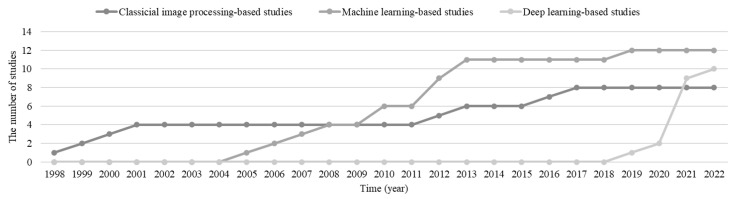
The increasing trends of the *Included Study* by method categories (the classical image processing-based studies, machine learning-based studies, and deep learning-based studies).

Figure 9 illustrates the accumulation trend of *Classical Image Processing-based Study*, *Machine Learning-based Study*, and *Deep Learning-based Study* in the *Included Study*. The specific publication date can be found in Table 2. The number of *Classical Image Processing-based Studies* increased in 1998, 1999, 2000, 2011, 2012, 2015, and 2016, while the slopes are slight. The number of *Machine Learning-based Studies* increased from 2004 to 2013, and the slopes are more significant. The number of *Deep Learning-based Studies* has rapidly increased since 2018. Figure 9 indicates that extending the machine learning and deep learning technologies to planetary exploration is delayed. The DBN was proposed in 2006, while the rapid related study attempted deep learning 11 years later (in 2018). Although the accumulated studies using deep learning are lower than for the *Machine Learning-based Study*, their increasing slope is significantly high. *Deep Learning-based Studies* caught up with the number of *Classical Image Processing-based Studies* in only four years. It is reasonable to expect that the *Deep Learning-based Study* can contribute further semantic terrain segmentation contributions.

The learning-based studies can be divided into supervised and unsupervised learning [95]. The training process of supervised learning depends on the difference between the prediction and ground-truth label, and the loss function measures their difference. In contrast, unsupervised learning is usually used in difficult-to-label cases. The unstructured environment in planetary exploration is difficult to label, which seems to be suited for unsupervised learning. Twenty-six percent of the *Included Study* used unsupervised learning (K-mean cluster and SVM), while their performance is not compatible enough with supervised learning (see Table 3 for the method of the *Included Study*). For example, the precision in reference [59] is only about 65%, while reference [65] achieved precision of more than 99% (see Table 3 for the quantitative results). Rock-based terrain segmentation is a typical difficult and unstructured environment, highly influenced by irregular and changing rock texture, size, and outline. Supervised learning requires many pixel-level labels, and manual annotation efficiency is low and human error is easily introduced. Therefore, it is promising to utilize transfer learning and weak supervision. For example, the transfer learning in reference [65] applied synthetic data to achieve prior knowledge, and then only little labeling is required to fine-tune the prior knowledge. Moreover, reference [67] utilized weak supervision to significantly decrease human error and labeling difficulty by using the proposed “conservative annotation method”, and cooperating with transfer learning.

#### 4.1.5. SubRQ5: What Data Have Been Used?

**Figure 10:**
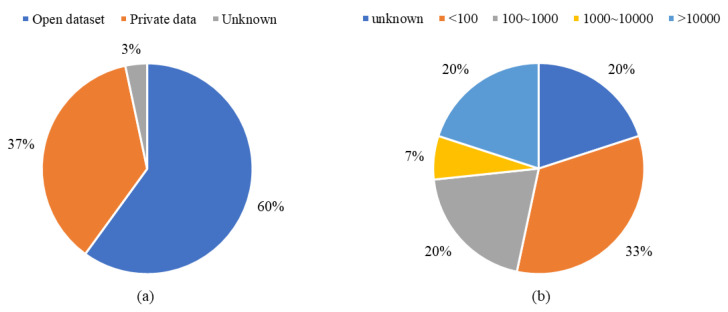
The distributions by dataset types and the number of images in the *Included Study*. (**a**) Refers to the distribution by dataset type, and (**b**) refers to the distribution by the number of images in the source data.

Data are one of the essential driving powers for artificial intelligence technologies [96]. Data are also an essential factor for research reimplementation. The “S_data_” and “N_img_” columns in Table 2 list the source data used and the number of images, respectively. Figure 10a uses a pie chart to classify the used dataset as the open dataset, private data, and unknown, and Table 11 indicates the study of the distributions by dataset types as in Figure 10a. The open dataset refers to the data available online, while the private data refers to the source data that are not available to the public. Sixty percent of data utilized in the *Included Study* were open-source data, or their source data are open. Figure 10b further analyzed the distribution by the number of images in the source data. Table 12 shows the distributions’ study by the number of images in Figure 10b. Fifty-three percent of open-source datasets have less than 1000 images, indicating that the available data for planetary exploration research is not much. Although past planetary rovers provided many images or videos, they are unlabeled raw data, which are difficult to use directly.

**Figure 11:**
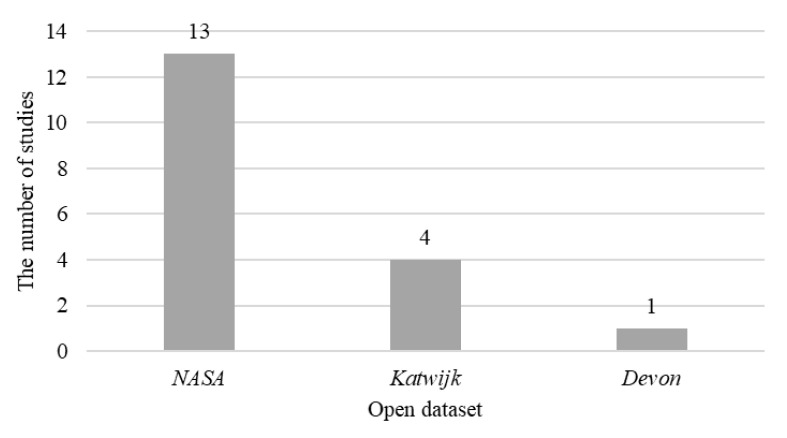
The number of open datasets in the *Included Study*. The “NASA”, “*Katwijk*”, and “*Devon*” refer to the NASA image set of Mars Exploration Rovers, the Katwijk beach planetary rover dataset, and the Devon Island rover navigation dataset, respectively.

Figure 11 illustrates the usage of the open datasets in the *Included Study*, and Table 13 depicts the study of the open datasets in Figure 11. The NASA image set [97] is the most popular dataset, while the European Space Agency (ESA) Katwijk beach planetary rover navigation dataset [98] is second. The data in the NASA image set are individual images, while the ESA Katwijk dataset provided the navigation video.

#### 4.1.6. SubRQ6: What Metrics Have Been Utilized for Evaluation?

The “Quanti.” column in Table 2 lists the evaluation metrics in the *Included Study*, including accuracy, precision, recall, Dice score (F1), IoU, and inference time. The qualitative results refer to the visualization, providing intuitive sense to readers. The “Quali.” column in Table 2 lists the situation of the qualitative results. The “Yes” and “No” refer to the qualitative results that are provided and not provided, respectively. Table 3 depicts the numerical results of the “Quanti” column in Table 2. It is noteworthy that the absolute values of these quantitative results are not comparable because they are achieved from different environments, source data, and experimental settings.

Equations (1)–(5) refer to accuracy, precision, recall, Dice score (F1), and IoU, respectively [95]. The character “N” refers to the number of samples in the corresponding category. The subscripts “TP”, “TN”, “FP”, and “FN” refer to true-positive, true-negative, false-positive, and false-negative categories, respectively. The “T” and “F” stand for “true” and “false” in the predictions, while “P” and “N” stand for “positive” and “negative” in the ground-truth labels. The accuracy represents the rate of correct predictions in all samples. The precision refers to the rate of correct true-predictions in the positive samples. The recall is the rate of correct (true and false) predictions in the positive samples. The IoU is a popular metric in image segmentation research.

(1) $$Accuracy=\frac{N_{TP}+N_{TN}}{N_{TP}+N_{TN}+N_{FP}+N_{FN}}$$

(2) $$Precision=\frac{N_{TP}}{N_{TP}+N_{FP}}$$

(3) $$Recall=\frac{N_{TP}}{N_{TP}+N_{FN}}$$

(4) $$ice\;score=2\times \frac{Precision\times Recall}{Precision+Recall}=\frac{2\times N_{TP}}{N_{TP}+N_{FP}+N_{TP}+N_{FN}}$$

(5) $$Intersection\;over\;Union\;\left(IoU\right)=\frac{N_{TP}}{N_{TP}+N_{FP}+N_{FN}}$$

Accuracy (Equation (1)) indicates correct prediction among all pixels, an overall indicator for terrain and background predictions. Precision (Equation (2)) indicates the correct ratio within the predicted terrain pixels, and recall (Equation (3)) indicates the correct ratio within terrain pixels in the ground truth. The Dice score (Equation (4)) uses one value to cover both recall and precision. Any small recall or precision can cause the Dice score to result in a large value. IoU (Equation (5)) can prevent a skew prediction that all predictions are terrain pixels to achieve high precision.

### 4.2. The Answer to the KeyRQ: What Achievements Do Computer Vision and Artificial Intelligence Bring to the Terrain Segmentation in the Navigation Vision of Planetary Rovers?

This study summarizes the answer to the KeyRQ into the following four attributes consisting of data, solution, application, and performance.

1) Three prior open datasets and four new datasets are found in the *Included Study* because of the promotion of computer vision and artificial intelligence, and the new datasets brought more inspiration and possibility to future studies. The prior datasets are the NASA image album [99], the ESA Katwijk beach planetary rover navigation dataset [98], and the Devon Island rover navigation dataset [100]. The newly proposed datasets refer to the conservative annotation dataset [65], the synthetic rock segmentation dataset [67], the generated OAISYS dataset [66], and the Mars-Seg dataset [70]. It is noteworthy that the newly proposed datasets all applied the prior datasets as sources to create new data.
2) The computer vision and artificial intelligence findings are widely utilized in the *Included Study* (see Table 3 for details). The K-mean cluster, Deeplab family, U-Net family, Mask-R-CNN family, and classical image processing algorithms made considerable contributions to the semantic terrain segmentation topic.
3) The *Included Study* is used for many practical applications. For example, reference [29] applied the K-mean cluster to ensure safe wandering for the planetary rover; reference [11] utilized Mask-R-CNN to support the Mars sample return mission; and reference [28] used the mean-shift algorithm for geological analysis.
4) The *Included Study* claimed that they achieved superior performance by applying different computer vision and artificial intelligence technologies. Table 3 describes the details from the perspective of metrics, while accuracy and IoU are the most used criteria for performance. For example, reference [67] achieved accuracy of 99.58% by applying the modified U-Net++; and reference [66] accomplished IoU for the sky region of 0.9066.

### 4.3. Challenges and Corresponding Research Gaps

The challenges for semantic segmentation in the navigation vision of planetary rovers are mainly located in the following three aspects.

1) Data with pixel-level annotation are insufficient. Although much data on planetary rover navigation vision exist, most are not annotated raw images or videos. It is difficult to use these images and videos effectively, considering the unsatisfactory performance of unsupervised and self-supervised solutions.
2) The pixel-level accuracy of semantic segmentation needs to be improved. The pixel-level accuracy refers to a broad idea, and there is no unified metric existent in current studies for evaluation. However, the pixel-level accuracy in most studies is not ideal, while pixel-level accuracy is significantly important for further functionalities based on semantic segmentation. For example, errors in obstacle contours can greatly affect the safety of path planning, and errors in rock detection may misjudge their hazards to wheels.
3) Third, existing methods lack discussion of real-time performance regarding onboard hardware. Some studies have obtained excellent segmentation accuracy, but they are all tested on offline hardware. The results of existing studies are still far from practical rover applications.

Therefore, the research gaps can come from the corresponding challenges:

1) An open, pixel-level annotated, and real-world image-based dataset is highly required, which may involve numerous efforts and time in data annotation.
2) Based on the open dataset, a unified metric for the evaluation benchmark is demanded, which can form a standard for comparison with related studies.
3) The onboard hardware test is essential for evaluating the practical performance of the corresponding solution.

### 4.4. Limitations of This Study?

The limitations of this systematic literature review came from the following three points from the guideline in references [33,34]:

1) The manual identification step is conducted in the identification step of the search process, which is recommended for software technology evaluation.
2) The candidate studies are identified by a single researcher, while the research questions, search strategy, exclusion criteria, and quality assessment are reviewed by other researchers.
3) The definitions of the “*Classical Image Processing-based Study*”, “*Machine Learning-based Study*”, and “*Deep Learning-based Study*” are only proposed in this study, which do not belong to common practice while highlighting the impact of technologies on terrain segmentation.

The first point indicates that some relevant research might not be identified by the search strategy. Especially, the studies are archived in national journals or conferences, and the studies are not written in English. Therefore, this study should be stick to a systematic literature review in the English-written major international journals and conferences.

The second point is implicit that the search field for different keywords might contain some bias. For example, regarding the keywords “terrain*”, “*rock*”, and “sky*”, the search fields of the IEEE Xplore, Web of Science, and Scopus are set to “All Metadata”, “AB (abstract)”, and “TITLE-ABS-KEY (title, abstract, and keywords)”, respectively. The three databases have different settings for the search field, and the decision of choosing the search field is decided by a single researcher and reviewed by another researcher.

The definitions of the “*Classical Image Processing-based Study*”, “*Machine Learning-based Study*”, and “*Deep Learning-based Study*” are introduced in Section 1 and Section 4.1.4. However, image processing, machine learning, and deep learning are not independent topics in common practice, which might not be precisely divided.

## 5. Conclusions

In summary, computer vision and deep learning have been making significant achievements in accurate navigation, intelligent geological analysis, and fast inference time through big data and artificial intelligence development. As planetary exploration projects progress worldwide [3,4,5], it is reasonable to look forward to further promising studies, attraction from global communities, and contributions via artificial intelligence.

This systematic literature review raises attention to the following five aspects:

i. Distributions: The community has increased interest in the semantic segmentation of navigation vision for planetary rovers. New studies are emerging significantly, and deep learning-based studies appear to have a significant increasing impact trend recently. However, the geological concentration is obvious, and the community’s interests have been considerably influenced by national space activities.
ii. Terrain targets: The rock is a challenging target with high value in geological analysis, navigation, and path planning. Although half of the *Included Study* addresses rock, an on-time rock segmentation solution with high pixel-level accuracy in onboard hardware does not exist yet.
iii. Open and annotated data: The discussion in Section 4.1.5 shows that more than half of the *Included Study* utilizes less than 1000 images, which is very abnormal considering the numerous data achieved in past space exploration projects. This review found that most space exploration data are raw and unannotated data, which are difficult to use directly. Thus, reference [13] proposes a large and annotated dataset (AI4Mars) to boost the research into planetary exploration. However, AI4Mars is a massive project that uses multiple labeler strategies, which is not a flexible strategy that can be broadcast to most topics (“multiple labelers” refers to the annotation conducted by more than one labeler, which can decrease human error.) Furthermore, AI4Mars does not result in the pixel-level annotations for the segmentation task, which is still a long way to the eventual semantic terrain segmentation for the planetary rovers. Therefore, the challenge for utilizing current raw and unannotated data can be specifically allocated to “how to annotate and efficiently use current data properly?” Furthermore, references [66,67,70] propose a synthetic algorithm for generating artificial images and annotations, but it is still challenging to justify the generalizability of synthetic data to the real world. Moreover, reference [65] proposes weak supervision to bypass the complicated annotation, but it can only work for large targets instead of small pixel globs (such as stones or sample tubes).
iv. Performance: Section 4.1.6 mentions that accuracy and efficiency are two widely used metrics in the *Included Study*. However, there are no standard metrics to evaluate the performance of the terrain segmentation solution. The *Included Study* applies various metrics according to their specific mission, which increases the difficulty of horizontal comparison for state-of-the-art.
v. Challenges: There is no existing dataset that is open to all communities, with a pixel-level annotation, and that uses real-world images. The significant challenges will be massive efforts of data annotation with minimum human error, which brings further difficulty to a standardized benchmark of state-of-the-art. The on-broad test for real-time evaluation will depend highly on the planetary rover platform, considering that only a few countries have the ability to produce planetary rovers. The solution will be to build an open environment for related research, which requires significant effort to construct an open dataset, a standard benchmark, and an online remote test platform for the community.

## Figures and Tables

**Table 1:** The research questions addressed by this systematic literature review.

Index	Content
KeyRQ	What achievements do computer vision and artificial intelligence bring to terrain segmentation in the navigation vision of planetary rovers?
SubRQ1	Why is terrain segmentation important for planetary explorations?
SubRQ2	What targets does current research pay attention to?
SubRQ3	What have visual sensors been applied to for obtaining data?
SubRQ4	What solution does current research have?
SubRQ5	What data have been used?
SubRQ6	What metrics have been utilized for evaluation?

The RQ, KeyRQ, and SubRQx refers to the research question(s), key research question, and sub-research question x, and these terminologies are used throughout the entire study.

**Table 2:** The details of the *Included Study*.

Ref.	Year	Terrain	F_data_	S_cate_	Algorithm	S_data_	N_img_	Quali.	Quanti.
[9]	1998	obstacles	depth	IP.	curve-based localization	customized data	2	No	Position accuracy, orientation accuracy
[54]	1999	rock	gray	IP.	Brodatz filter	customized data	N/A	Yes	No
[55]	2000	obstacles	gray	IP.	illumination and pixel height	NASA Mars	100	No	*S_AB_* (the intersection of segmentations *A* and *B* divided by the union of the two segmentations)
[56]	2001	terrains	gray	IP.	Feature extraction and fuzzy logic	customized data	N/A	Yes	No
[57]	2005	rock	RGB	ML.	K-mean cluster	customized data	30	No	Precision, recall, localization
[58]	2006	rock	gray	ML.	SVM	NASA Mars Exploration Rover “*Spirit*”	1	Yes	No
[59]	2007	rock	gray	ML.	K-mean cluster	NASA Mars Exploration Rover “*Spirit*”	150	Yes	Accuracy, recall, precession, and chamfer distance
[30]	2008	rock	RGB	ML.	K-mean cluster	N/A	N/A	Yes	No
[29]	2010	rock	gray	ML.	K-mean cluster	customized data	N/A	No	No
[14]	2010	rock	gray	ML.	K-mean cluster	customized data	N/A	Yes	No
[60]	2011	terrains	N/A	ML.	auto-regressive (AR) model	customized data	300	No	Euclidean distance, Martin’s distance, KDF on the Stiefel manifold
[61]	2012	terrains	N/A	ML.	auto-regressive (AR) model	customized data	300	No	Kullback–Leibler (K-L) distance, cepstral distance, the distance based on the feature vector
[26]	2012	rock	gray	ML.	Superpixel and K-mean cluster	NASA Moon image set	40	Yes	Inference time, accuracy, boundary error, precision, and recall
[62]	2013	skyline	gray	ML.	Canny, SIFT, and SVM	customized data	10	Yes	Time and accuracy
[26]	2013	rock	gray	IP.	OTSU algorithm, Canny, and TDEL	customized data	1	No	No
[28]	2013	rock	gray	IP.	Mean-shift algorithm	NASA Mars	10	Yes	No
[31]	2013	sky	gray	DL.	K-mean cluster	NASA Mars	1,482	Yes	True-positive rate (TPR), false-positive rate (FPR), and receiver operating characteristic (ROC)
[63]	2016	rock	gray	DL.	Canny operator and regroup	NASA Mars	14	Yes	Time, memory footprint
[27]	2017	skyline	gray	ML.	Sobel and a multivariable thresholding method	NASA Mars “*Opportunity*”	243	Yes	Customized criteria: Good, okay, or poor
[32]	2019	rock	RGB	DL.	modified U-Net	Devon dataset	400	Yes	F-score and parameters
[10]	2019	rock	gray	DL.	gradient-region constrained level set method, evolution function, and minimization of the overall energy functional using the standard gradient descent method	NASA Mars	N/A	Yes	No
[64]	2020	rock	RGB	DL.	Deeplabv3+	ESA Katwijk	50	Yes	Accuracy and IoU
[11]	2021	sample tube	RGB	DL.	Mask-R-CNN	customized data	824	Yes	Average precision (AP), average recall (AR), precision-recall (PR) curves
[65]	2021	sky and ground	RGB	DL.	NI-U-Net	ESA Katwijk	22,000	Yes	Accuracy, precision, recall, dice score (F1), misclassification rate (MCR), root mean squared error (RMSE), and intersection over union (IoU)
[13]	2021	terrains	gray	DL.	Deeplabv3+	NASA Mars rover images	35,000	No	Accuracy
[66]	2021	obstacles	RGB	DL.	Mask-R-CNN	NASA Mars data and OAISYS generated data	many	Yes	IoU
[67]	2021	rock	gray	DL.	NI-U-Net++	ESA Katwijk	14,000	Yes	Accuracy, IoU, dice score, RMSE
[68]	2021	terrains	RGB	DL.	FCNN	NASA Curiosity	32,000	Yes	Confusion matrix
[69]	2021	obstacles	gray	DL.	Deeplabv3+	ESA Katwijk	22,000	Yes	Accuracy and IoU
[70]	2022	terrains	gray	DL.	modified Deeplabv2	NASA Mars data and Mars-Seg dataset	5000	Yes	Mean Intersection over Union (mIoU)

**Table 3:** The applied methods and quantitative results of the *Included Study*.

Ref.	Method	Quantitative Results
[29]	K-mean cluster	Not given
[26]	Super-pixel with entropy rate-based segmentation	(1) Speed = 12 s/image (5 super-pixels); (2) Accuracy = 81.95% (26 images have accuracy more than 80%); (3) Average boundary error = 13.34 pixels (29 images have error less than 15 pixels); (4) Precision = 0.25–1; (5) Recall = 0.45–1.
[64]	Deeplabv3+	Semantic terrain segmentation is only a part of the entire study. Some indirect results in the eventual 3D semantic map: (1) Data01: Accuracy = 96% and IoU = 0.58; (2) Data02: Accuracy = 90% and IoU = 0.21; (3) Data03: Accuracy = 95% and IoU = 0.36.
[59]	SVM	(1) Accuracy = 97.3%; (2) The standard deviation of Recall = 0.2–0.3; (3) the standard deviation of Precision = 0.2–0.3.
[11]	Mask-R-CNN	(1) Average precision = 0.918; (2) Average recall [0.5:,0.05:,0.95] = 0.575.
[30]	K-mean cluster and histogram	Not given
[27]	Sobel operator and gradient-based multi-threshold horizontal detection	(1) Default threshold: 88.9%; (2) Adjust threshold: 98.3%.
[56]	Feature extraction and roughness calculation	Not given
[13]	Deeplabv3+	(1) NavCam-Merged: Accuracy = 96.67%; Individual accuracy = Soil: 96.00%, Bedrock: 90.87%, Sand: 96.51%, Big rock: 82.83%. (2) NavCam-Random: Accuracy = 94.97%. Soil: 99.10%, Bedrock: 94.90%, Sand: 93.45%, Big rock: 93.24%.
[57]	K-mean cluster and Beyers network	(1) Precision = 0.65–0.87; (2) Recall = 0.05–0.72; (3) Localization = 0.17–0.42.
[9]	Curve-based localization	(1) Position accuracy = 5 and 20 cm; (2) Orientation accuracy = 5 degree.
[55]	Altitude and brightness	(1) *S_AB_* = (manual) 0.68; (2) *S_AB_* = (automatic) 0.34.
[54]	Brodatz filter	Not given
[28]	Mean-shift algorithm	Not given
[62]	Canny, SIFT, and SVM	(1) Dynamic Programming (DP) and Canny: Time = 14.35–39.49 s; Accuracy = (total error percentage) 0.07–72.50%; (2) DP and Maximally Stable Extremal Edges (MSEE): Time = 22.96–43.87 s; (3) Accuracy = (total error percentage) 0.07–51.16%.
[63]	Canny operator	(1) Time = 600–900s (image resolution is 1000 × 1000); (2) Maximum memory footprint = 4 MB.
[31]	K-mean cluster and neural networks	(1) TPR (True-Positive rate) = 0.9886; (2) FPR (False-Negative rate) = 4.0461 × 10^−4^.
[32]	Modified U-Net	F-score = 78.5% (1,939,170parameters).
[10]	Gradient-region constrained level set method, evolution function, and minimization of the overall energy functional using the standard gradient descent method	Not given
[65]	Modified U-Net (NI-U-Net)	(1) Accuracy = 99.232%; (2) Precision = 99.211%; (3) Recall = 99.221%; (4) Dice score (F1) = 99.104%; (5) Misclassification rate (MCR) = 0.0077; (6) Root mean squared error (RMSE) = 0.0427; (7) Intersection over union (IoU) = 0.98223.
[71]	OTSU algorithm, Canny operator, and template dilatation edge linking (TDEL)	Not given
[61]	Autoregressive (AR) model	(1) Kullback–Leibler (K-L) dist.: 96.4%; (2) Cepstral dist.:94.6%; (3) The dist. based on the feature vector: 93.5%.
[60]	Autoregressive (AR) model	(1) Dynamic texture: Euclidean dist. = 89.9–94.5%; Martin’s dist. = 91.1–96.3%; KDF on the Stiefel manifold = 0.4–70.8%. (2) Static texture: Euclidean dist. = 97.8–99.5%; Martin’s dist. = 99.3–99.9%; KDF on the Stiefel manifold = 81.8–97.6%.
[14]	K-mean cluster	Not given
[58]	K-mean cluster	Not given
[66]	Mask-R-CNN	(1) IoU (sand) = 0.8139; (2) IoU (gravel) = 0.8138; (3) IoU (Dried mud) = 0.4117; (4) IoU (sky) = 0.9066.
[70]	Modified deeplabv2	mIoU (ISPRS dataset) = 54.34; mIoU (Aerial) = 48.25; mIoU (Mars-Seg) = 64.76.
[67]	Modify U-Net++ (NI-U-Net++)	Katwijk dataset: (1) Accuracy = 99.58%; (2) IoU = 74.76; (3) Dice = 0.8556; (4) RMSE = 0.0557.
[68]	Fully convolutional neural networks (FCNN)	(1) Confusion matrix; (2) Accuracy (loose sand) = 62.6%; (3) Accuracy (small rocks) = 88.6%; (4) Accuracy (bedrock) = 68.1%; (5) Accuracy (outcrop) = 56.4%; (6) Accuracy (large rocks) = 73.2%; (7) Accuracy (sand dune) = 45.3%; (8) Accuracy (dense ridge) = 83.7%; (10) Accuracy (background) = 94.8%.
[69]	Deeplabv3	Accuracy = 98.7%; IoU = 0.282.

The “Ref.” refers to the reference index. The “Method” column describes the method used in the corresponding study, and many deep learning methods are modified from recent studies. The following is the documentation of these existing studies in favor of further exploration. The details of “Deeplabv2”, “Deeplabv3+”, “Mask-R-CNN”, U-Net, U-Net++, and “Fully convolutional neural networks (FCNN)” can be found in references [46,72,73,74,75,76], respectively.

**Table 4:** The geographical distribution by location (country and continent) in the *Included Study*.

Country	USA	Mexico	UK	Italy	Germany	France	China	Japan
The number of studies	13	1	2	2	1	1	8	2
Continent	North America		Europe				Asia	
The number of studies	14		6				10	

**Table 5:** The reference list of the distribution by the experimental setting.

The Condition of the Experimental Setting	Ref.
Provided	[13,14,26,29,32,55,59,62,63,65,66,67,68,70,71]
Not provided	[9,10,11,27,28,30,31,54,57,58,60,61,64,69]

The “Ref.” column refers to the reference index of the study.

**Table 6:** The results of the quality assessment in the *Included Study*.

Ref.	Theory Robustness	Implication for Practice	Methodology, Data Supporting Arguments	Generalizability	Contribution Plus a Short Statement Summarizing the Article’s Contribution	Sum in Points
[29]	2	2	2	1	1	8
[26]	2	2	3	3	3	13
[64]	2	2	2	2	2	10
[59]	3	3	3	3	3	15
[11]	3	3	3	3	1	13
[30]	2	3	3	3	1	12
[27]	2	2	3	2	1	10
[56]	2	2	2	2	1	9
[13]	3	3	3	3	3	15
[57]	1	2	3	2	1	9
[9]	2	2	2	2	1	9
[55]	1	2	2	2	1	8
[54]	3	2	2	2	1	10
[28]	3	2	2	2	2	11
[77]	1	2	1	1	1	6
[62]	3	2	2	2	1	10
[63]	2	3	3	3	1	12
[31]	2	2	2	2	1	9
[32]	2	3	2	3	1	11
[84]	1	1	2	1	1	6
[10]	3	3	2	2	1	11
[85]	0	2	2	2	1	7
[65]	3	2	3	2	3	13
[71]	3	2	2	2	2	11
[61]	2	2	2	1	1	8
[60]	2	2	2	1	1	8
[14]	2	2	2	1	1	8
[58]	2	2	2	2	1	9
[66]	3	3	3	3	3	15
[70]	3	3	3	3	3	15
[67]	3	3	3	2	3	14
[68]	3	3	3	3	3	15
[69]	3	2	3	2	3	13

The “Ref.” refers to the reference index. The “Theory robustness”, “Implication for practice”, “Methodology, data supporting arguments”, “Generalizability”, and “Contribution plus a short statement summarizing the article’s contribution” columns apply the quality assessment criteria in reference [51]. The “0”, “1”, “2”, “3”, and “Not applicable” refer to the level of absence, low, medium, high, and not applicable, respectively. The “Sum in points” column refers to the accumulated points of the five quality assessment items. The shading rows refer to the excluded studies by the quality evaluation.

**Table 7:** The classification of the field of application in the *Included Study*.

The Field of Application	Ref.
Navigation	[9,13,14,26,29,31,55,60,61,62,65,66,67,68,69,71,85]
Geological analysis	[10,26,28,30,32,58,70]
Exploration efficiency	[13,27,54,55,57,59,63,64,65,67]
Other particular purposes	[10,11,54]

The “Ref.” refers to the reference index.

**Table 8:** The reference list to corresponding targets in Figure 6.

Target	Ref.
Sample tube	[11]
Terrains (include craters)	[13,56,60,61,68,70]
Obstacle	[9,55,66,69]
Skyline	[27,62]
Sky and ground	[31,65]
Rock	[10,14,26,28,29,30,31,54,57,58,59,63,64,67,71]

The “Ref.” column refers to the reference index of the study.

**Table 9:** The study of the distribution by camera model in Figure 7a.

Camera Model	Ref.
Stereo	[9,14,29,55,64,69]
Mono	[10,11,13,26,27,28,30,31,32,54,56,57,58,59,60,61,62,63,65,66,67,68,70,71]

**Table 10:** The study of the distribution by image format in Figure 7b.

Image Format	Ref.
Grayscale image	[10,13,14,26,27,28,29,31,54,55,56,58,59,61,62,63,67,70,71]
Color image	[11,30,32,57,64,65,66,68]
Depth image	[9]
Unknow	[60,69]

The “Ref.” column refers to the reference index of the study.

**Table 11:** The study of the distributions by dataset types in Figure 10a.

Dataset Type	Ref.
Open dataset	[13,26,27,28,31,32,55,58,63,64,65,66,67,68,69,70]
Private data	[9,11,14,29,54,56,57,60,61,62,71]
Unknown	[30]

The “Ref.” column refers to the reference index of the study.

**Table 12:** The study of the distributions by the number of images in Figure 10b.

The Number of Images	Ref.
unknown	[10,14,29,30,54,56]
≤100	[9,26,28,55,57,58,63,64,71]
100–1000	[11,27,32,59,60,61]
1000–10,000	[31,70]
>10,000	[13,65,66,67,68,69]

The “Ref.” column refers to the reference index of the study.

**Table 13:** The study of the open datasets in Figure 11.

Open Dataset	Ref.
NASA	[10,13,26,27,28,31,55,58,59,63,66,68,70]
Katwijk	[64,65,67,69]
Devon	[32]

The “Ref.” column refers to the reference index of the study.

## Appendix A

This section lists similar surveys by partially expanding the search keywords discussed in Section 2.2. Table 2 and Table 3 include all related studies in the three databases (IEEE Xplore, Scopus, and Web of Science), while Table A1 lists similar surveys related to this SLR. It is noteworthy that all related surveys in Table A1 are found in the Scopus database, and only the surveys published in the past three years are considered to maintain its advances.

The four search commands in Section 2.2 are (ii-a), (ii-b), (iii-a), and (iii-b), and the new search command is “TITLE (review OR survey)” to specify the type to be surveyed. There are no surveys found by combining (ii-a), (ii-b), (iii-a), (iii-b), and the new search command, no surveys found by combining (ii-b), (iii-a), (iii-b), and the new search command, three surveys (refs. [101,102,103]) found by combining (ii-a), (iii-a), (iii-b), and the new search command, two surveys (refs. [104,105]) found by combining (ii-a), (ii-b), (iii-b), and the new search command, and no surveys found by combining (ii-a), (ii-b), (iii-a), and the new search command.

**Table A1:** The comparison between this SLR and similar surveys.

Ref.	The Difference Compared to This SLR
[101]	This survey is not an SLR. This survey did not concentrate on the impact of computer vision and artificial intelligence. This survey studied the Martian and lunar lava tube instead of the terrain for planetary rover navigation.
[102]	This survey is not an SLR. This survey did not concentrate on the impact of computer vision. This survey studied the characterization of planetary soils instead of the terrain for planetary rover navigation.
[103]	This survey is not an SLR. This survey studied the autonomous spacecraft relative navigation technology instead of the terrain for planetary rover navigation.
[104]	This survey is not an SLR. This survey did not concentrate on the impact of computer vision and artificial intelligence. This survey studied the transmissive fractures in crystalline aquifer instead of the terrain for the planetary rover navigation.
[105]	This survey is not an SLR. This survey focused on information fusion instead of the impact of computer vision and artificial intelligence. This survey studied the GNSS and on-board vision-based solution of environmental context detection instead of the terrain for the planetary rover navigation.

Ref. refers to the reference index.
